# Synthesis, characterization and crystal structure of methyl 2-(2-oxo-2*H*-chromen-4-yl­amino)­benzoate

**DOI:** 10.1107/S2056989023007351

**Published:** 2023-08-29

**Authors:** Henrique V. P. Hollauer, Rachel C. Vilas Novas, Guilherme P. Guedes, Camilla D. Buarque, Lívia B. L. Escobar

**Affiliations:** a Chemistry Department, Pontifical Catholic University of Rio de Janeiro, 22451-900 Rio de Janeiro, RJ, Brazil; b Chemistry Institute, Federal Fluminense Universidade, Niteroi, 24020-141 Rio de Janeiro, Brazil; Tulane University, USA

**Keywords:** crystal structure, coumarin, benzoate, ester

## Abstract

Methyl 2-(2-oxo-2*H*-chromen-4-yl­amino)­benzoate, C_17_H_13_NO_4_ (**1**), was pre­pared by condensation between 4-hy­droxy­coumarin and methyl 2-amino­benzoate. It crystallizes in the ortho­rhom­bic space group *Pca*2_1_ at 300 K. The mol­ecule of compound **1** consists of the 2*H*-chromen-2-one part connected by an amine moiety (–NH–) to the methyl benzoate ring. The supra­molecular array is formed by hydrogen bonds between the aromatic ring and the O atoms of the lactone and ester portions. The structural details match the spectroscopic data acquired from NMR and IR spectroscopy.

## Abstract 2

A new coumarin derivative, methyl 2-(2-oxo-2*H*-chromen-4-yl­amino)­benzoate, has been synthesized and characterized.

## 1. Chemical context

**Figure 1:**
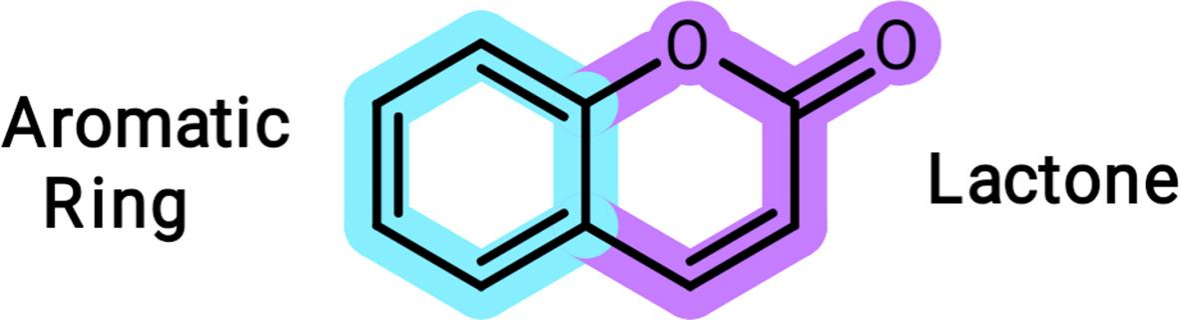
The main structure of coumarins.

Coumarins are an important class of lactones composed of benzene fused to an α-pyrone ring (Fig. 1 ▸). These structures have two pharmacophoric groups: the aromatic ring, which can promote hydro­phobic inter­actions, such as π-inter­actions, and the lactone group, which is a hydrogen-bond acceptor with receptors such as enzymes (Yildirim *et al.*, 2023 ▸).

These compounds are widely distributed in nature, especially as secondary metabolites of vascular plants. Coumarin was first isolated from tonka beans (*Dipteryx odorata* Wild; Fabaceae family) by Vogel in 1820. Since then, more than 1300 coumarins have been identified from natural sources (Bor *et al.*, 2016 ▸).

Their versatile scaffold also brings a wide range of applications, such as biocides, phytochemicals, pharmacological agents and flavorings, widely used in different industries. In medicinal chemistry, a widely used coumarin drug is warfarin, an anti­coagulant that has made it possible for thrombosis treatment to be done orally (Annunziata *et al.*, 2020 ▸). In addition, multiple biological activities are well known, including anti-inflammatory (Bansal *et al.*, 2013 ▸), anti­microbial (Regal *et al.*, 2020 ▸), anti­oxidant (Rosa *et al.*, 2021 ▸), anti-allergic (Liu *et al.*, 2019 ▸), anti-HIV (Xu *et al.*, 2021 ▸), anti­cancer (Emam *et al.*, 2023 ▸) and anti­viral (Sharapov *et al.*, 2023 ▸) activities.

**Figure 2:**
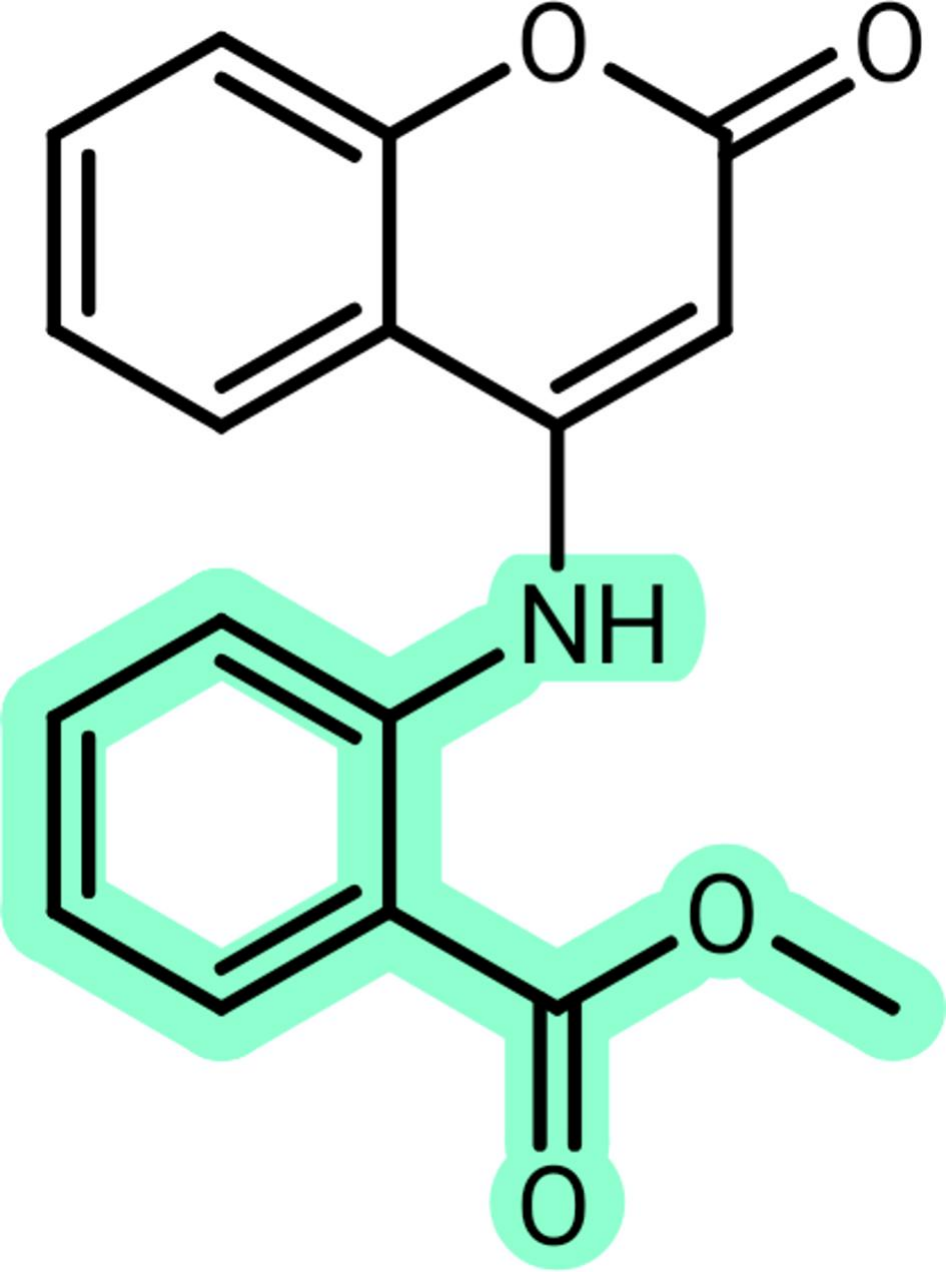
Chemical structure of **1**.

Recent work has demonstrated the importance of cou­marins in the design of small-mol­ecule fluorescent chemosensors (Cao *et al.*, 2019 ▸). Here we report the synthesis and characterization of methyl 2-(2-oxo-2*H*-chromen-4-yl­amino)­benzoate, **1** (Fig. 2 ▸), by condensation between 4-hy­droxy­coumarin and methyl 2-amino­benzoate, according to the literature (Carneiro *et al.*, 2021 ▸). The principal purpose of producing this compound was to investigate its biological properties because coumarin derivatives are potential candidates for anti­leishmaniasis drugs (Carneiro *et al.*, 2021 ▸). Also, studies involving the complexation of this mol­ecule with metal ions, such as Cu^II^ and Gd^III^, are in progress in our laboratory for future contributions.

## 2. Structural commentary

**Figure 3:**
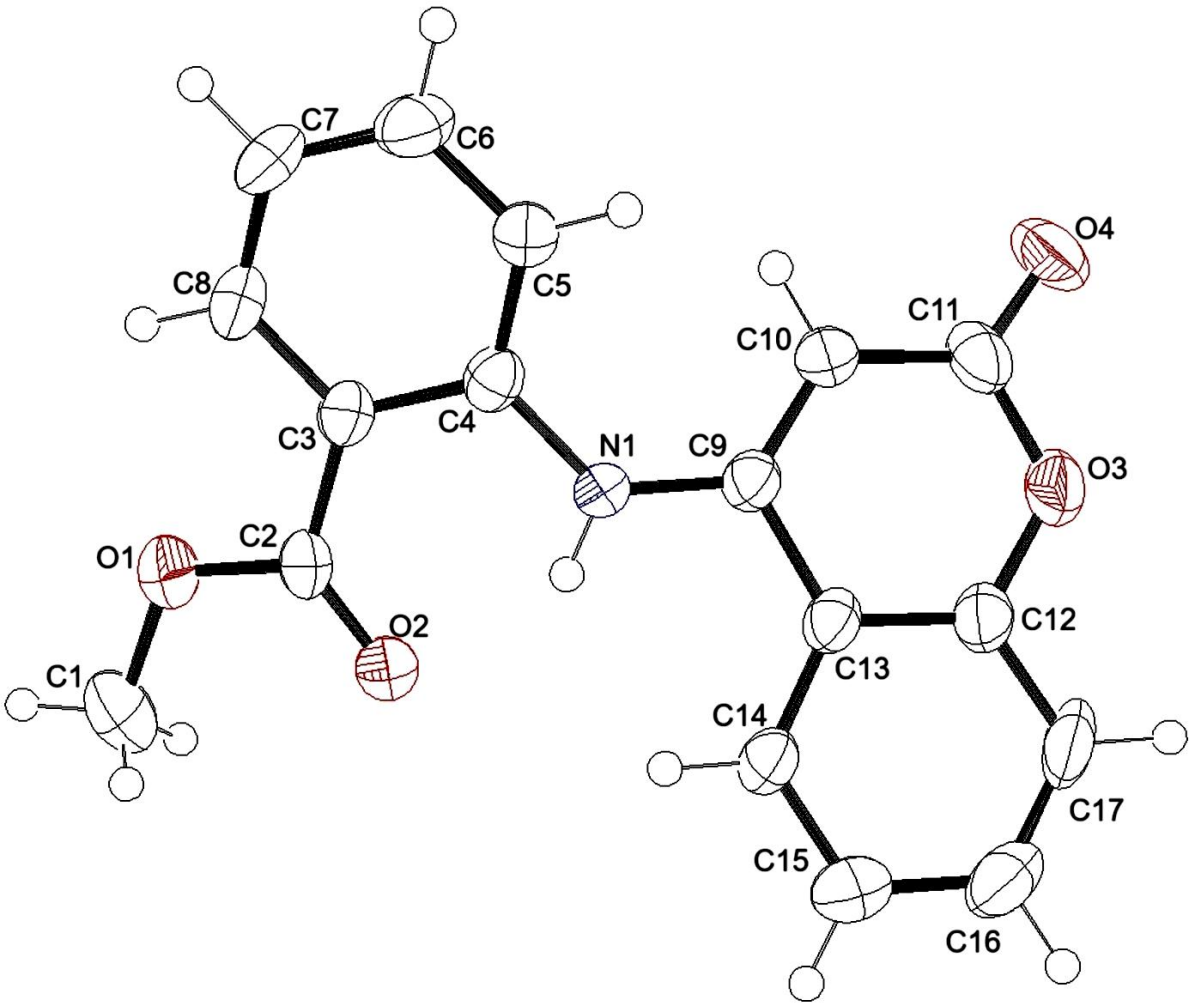
The asymmetric unit of **1**, with the numbering scheme and 50% probability displacement ellipsoids.

Compound **1** was synthesized *via* a reaction of the precursor coumarin and the corresponding aniline (Scheme 1). The resulting compound was recrystallized from di­methyl­formamide to yield yellow single crystals. Compound **1** crystallizes in the ortho­rhom­bic space group *Pca*2_1_, with the asymmetric unit consisting of one methyl 2-(2-oxo-2*H*-chromen-4-yl­amino)­benzoate mol­ecule (Fig. 3 ▸). The absolute structure could not be established with certainty. 
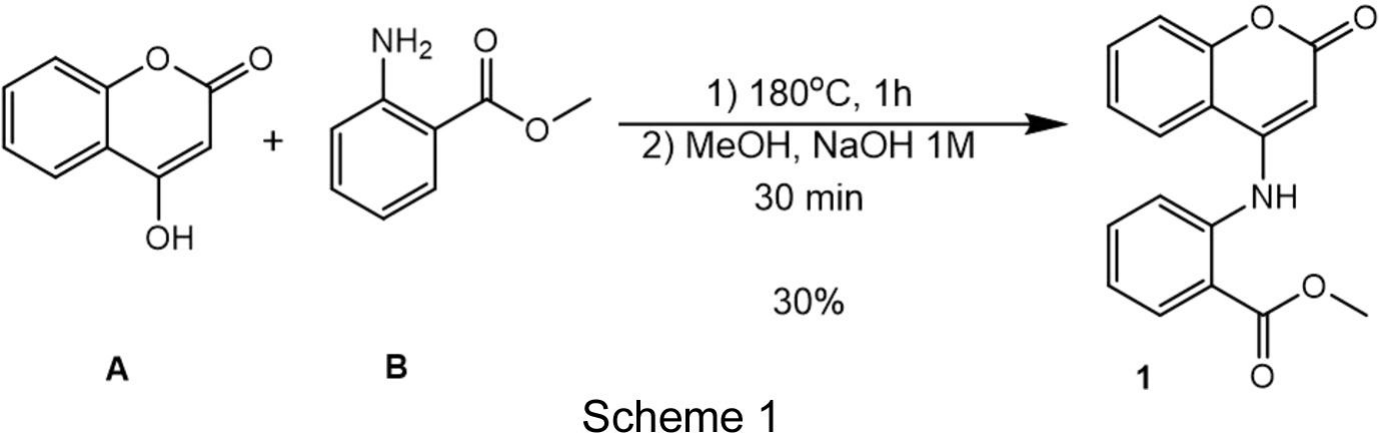


The average C—C bond distance in the aromatic portion of the coumarin is 1.374 (7) Å, while the C9—C13, C9—C10 and C10—C11 bond lengths in the lactone portion are 1.450 (7), 1.353 (7) and 1.412 (7) Å, respectively, because of the partial localization of π-bonding within the ring. The C11—O3 and C12—O3 bond lengths are equivalent at 1.374 (7) and 1.373 (7) Å, respectively, while the C11=O4 distance is 1.204 (7) Å. The sum of the angles about N1 is 359 (3)°, implicating involvement of its lone pair in N—C π-bonding. This is supported by the N1—C9 and N1—C4 distances of 1.351 (6) and 1.391 (6) Å, respectively. Similar geometrical parameters are found in closely related structures (see *Database survey* section), although the C4—N1—C9 angle at 130.9 (4)° is about 7° larger than in those mol­ecules, presumably due to the intra­molecular N1—H1⋯O2 hydrogen bond (Table 1 ▸). In the C3–C8 ring, the average C—C bond distance is 1.379 (8) Å, with the ester portion bond lengths of C2=O2 = 1.203 (6), C2—O1 = 1.316 (7) and C1—O1 = 1.440 (8) Å.

The dihedral angle between the mean plane which contains the main structure of the coumarin and the mean plane containing the aromatic ester portion is 31.21 (10)°.

**Figure 4:**
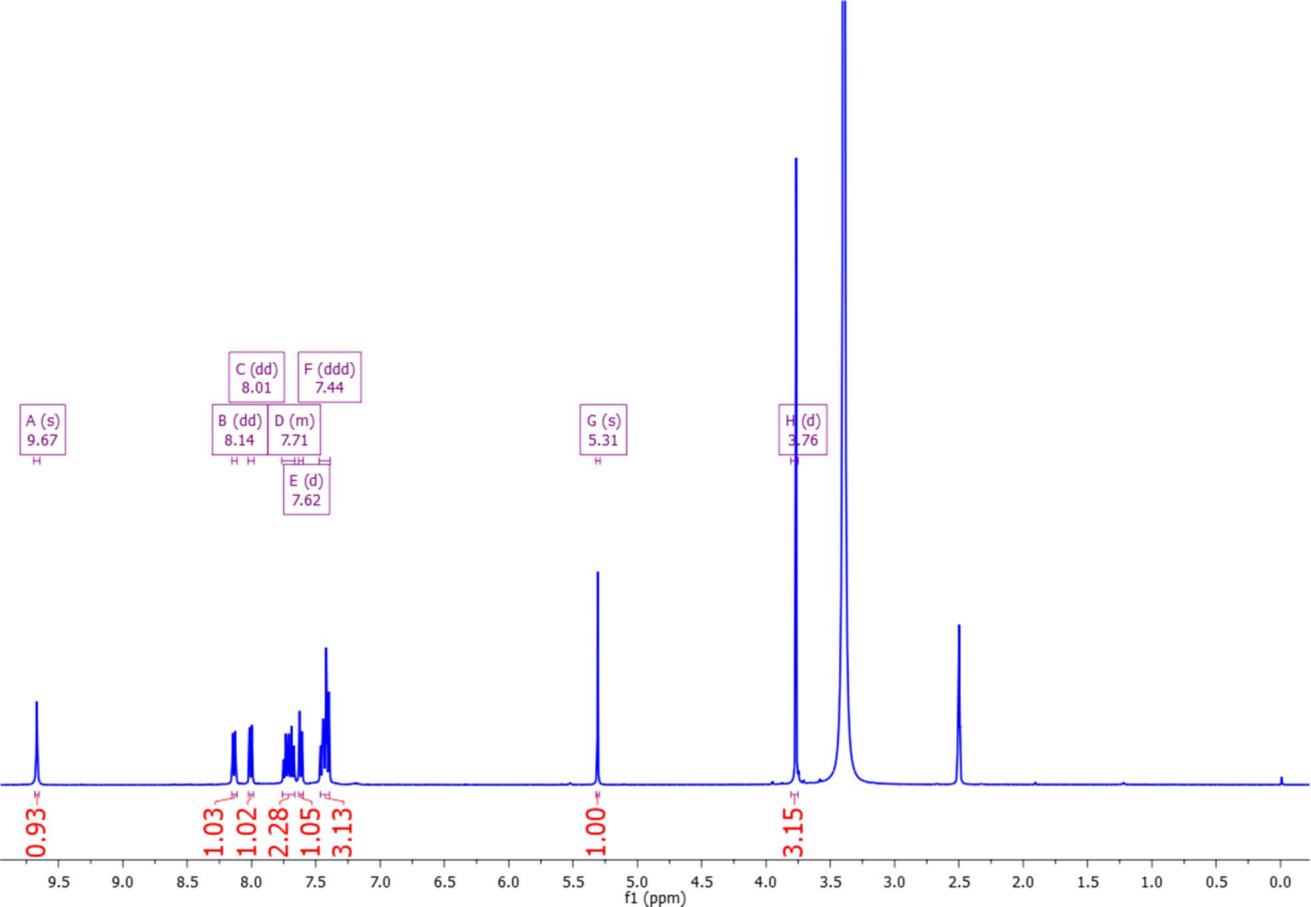
NMR-H: ^1^H NMR (400 MHz, DMSO-*d*_6_) δ 9.67 (*s*, 1H), 8.14 (*dd*, *J* = 8.1, 1.2 Hz, 1H), 8.01 (*dd*, *J* = 7.9, 1.5 Hz, 1H), 7.76–7.66 (*m*, 2H), 7.62 (*d*, *J* = 7.4 Hz, 1H), 7.44 (*ddd*, *J* = 15.4, 9.8, 4.6 Hz, 3H), 5.31 (*s*, 1H), 3.76 (*d*, *J* = 7.7 Hz, 3H).

The NMR spectra are shown in Figs. 4 ▸ and 5 ▸. The characterization by ^1^H and ^13^C NMR confirms the product as methyl 2-(2-oxo-2*H*-chromen-4-yl­amino)­benzoate. In the ^1^H NMR spectrum, there is a singlet at δ 3.74 ppm attributable to the meth­oxy group of the ester, the coumarin vinylic H atom appears at δ 5.31 ppm and a singlet is seen at δ 9.67 which can be assigned to N—H. In addition, there are eight aromatic H atoms between δ 7.44 and 8.14 ppm. In the ^13^C NMR spectrum, the meth­oxy group appears at δ 52.47 ppm, the two carbonyl C atoms at δ 166.50 and 161.40, and the vinylic and aromatic C atoms between δ 114 and 154 ppm.

## 3. Supra­molecular features

**Figure 6:**
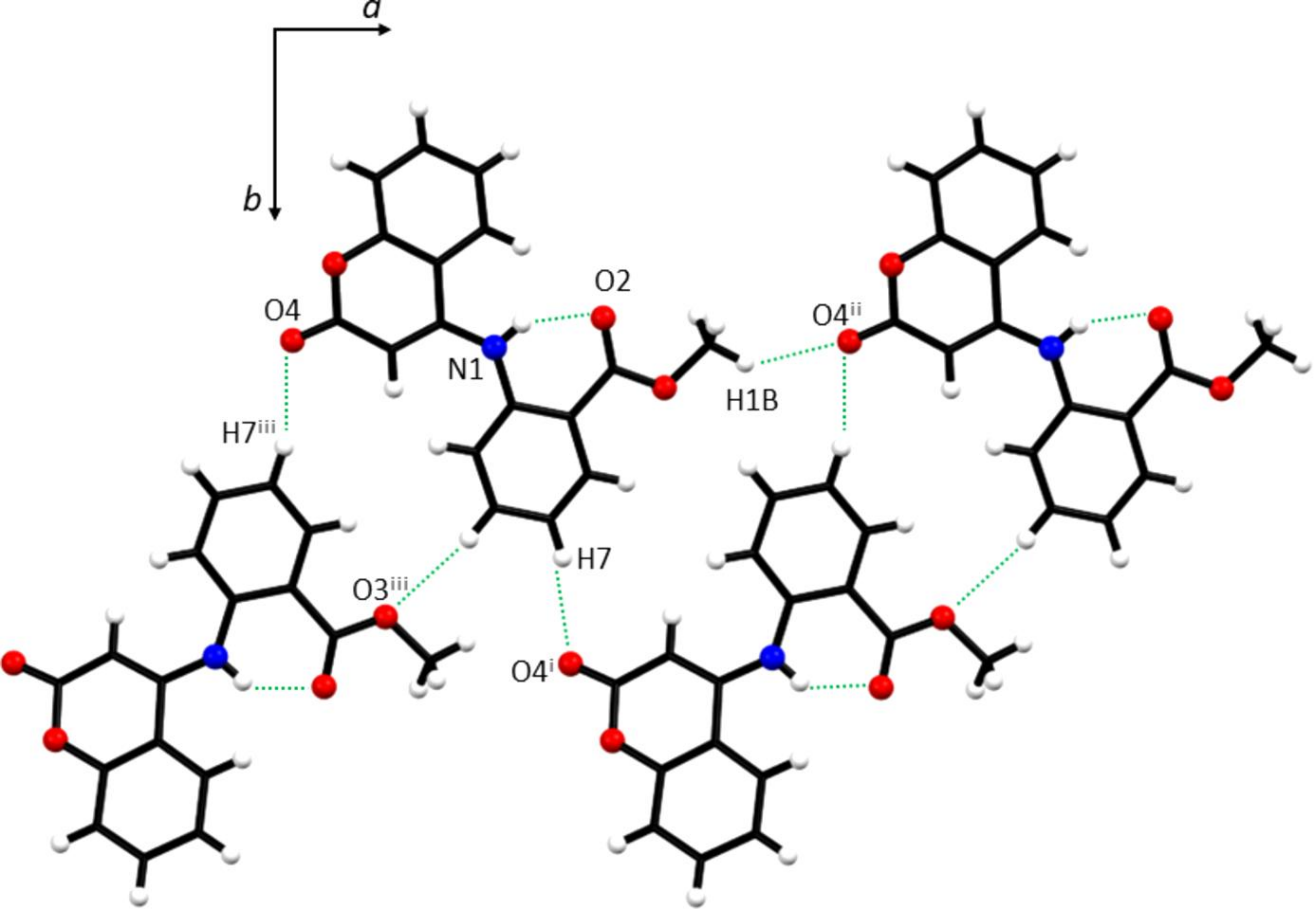
Supra­molecular array of **1**. [Symmetry codes: (i) *x* − 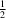 , −*y* + 2, *z*; (ii) *x* − 1, *y*, *z*; (iii) *x* + 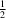 , −*y* + 2, *z*.]

**Figure 7:**
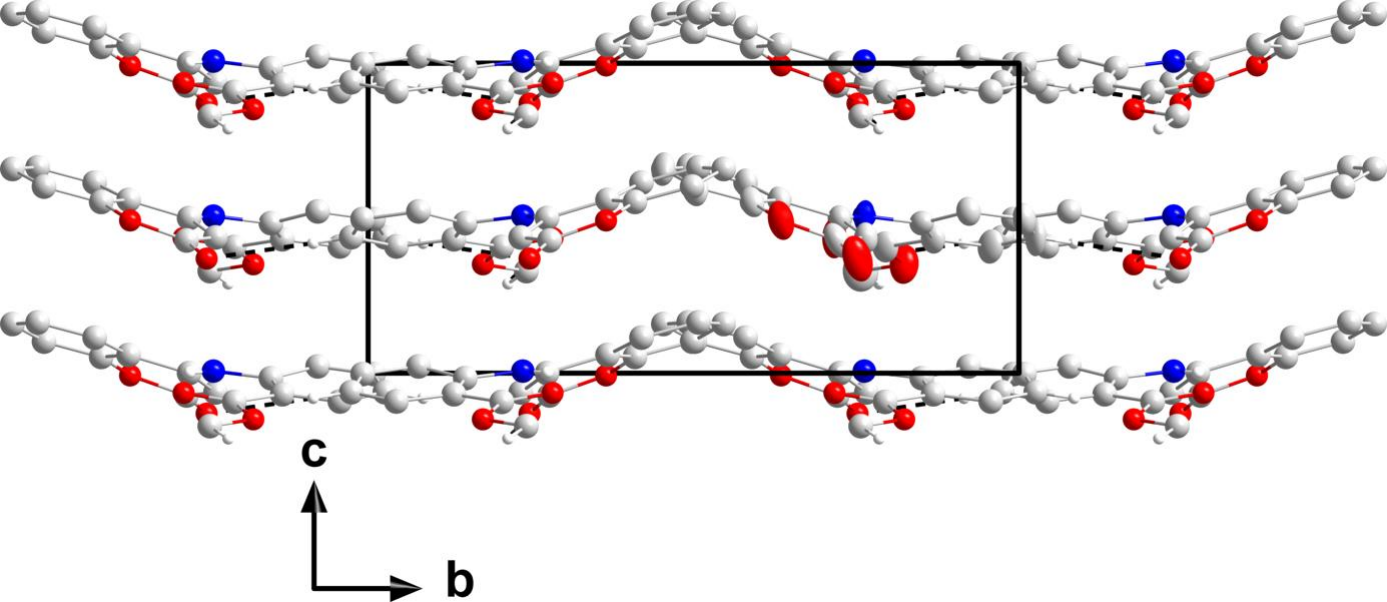
Packing viewed along the *a*-axis direction. The C—H⋯O hydrogen bonds are depicted by dashed lines and non-inter­acting H atoms have been omitted for clarity.

The supra­molecular array is formed by hydrogen bonds between the H atoms of the methyl group and the O atom of the lactone portion (C1—H1*B*⋯O4^i^) and the H atom from the aromatic ring (C7—H7⋯O4^ii^) (Table 1 ▸). These build corrugated chains two mol­ecules wide extending along the *a*-axis direction (Fig. 6 ▸). The crystal packing (Fig. 7 ▸) involves layers of chains parallel to the *ab* plane which stack along the *c*-axis direction, all associated through van der Waals inter­actions.

## 4. Database survey

A search of the Cambridge Structural Database (CSD; Groom *et al.*, 2016 ▸; updated to March 2023) yielded a substantial number of hits for chromenes having a nitro­gen-containing substituent in the 3-position of the lactone ring but relatively few with this substituent in the 4-position. Most of the latter also contained a second substituent in the 3-position, such as 4-[(4-bromo­phen­yl)amino]-3-(phenyl­selan­yl)-2*H*-chromen-2-one (OFIHOE; Belladona *et al.*, 2023 ▸), but only three are directly comparable to **1**. These are 4-(propyl­amino)-2*H*-chromen-2-one (HIDYEB; Kumar *et al.*, 2018 ▸), 4-[(pyridin-3-ylmeth­yl)amino]-2*H*-chromen-2-one (TUWLUV; Ait-Ram­dane-Terbouche *et al.*, 2020 ▸) and 4-(benzyl­amino)-2*H*-chromen-2-one (ZOKVIE; Campbell *et al.*, 1995 ▸). All three have structural parameters very similar to those of **1**, including essentially planar chromene portions and some localization of π-bonding in the lactone portion. The largest difference is seen for the exocyclic C—N—C angles which are around 123°.

## 5. Synthesis and crystallization

The reaction was carried out according to the literature (Carneiro *et al.*, 2021 ▸) (Scheme 1). A mixture of **A** and the aniline **B** (2 equiv.) was heated in a 50 ml Becher at 453 K for 1 h. A solution comprised of 30 ml of hot methanol and 30 ml of aqueous NaOH (1 mol l^−1^) was then added to the solid. This mixture was stirred for 30 min at 333 K and then filtered. The solid was washed with water, dried and used without further purification.

## 6. Refinement

Crystal data, data collection and structure refinement details are summarized in Table 2 ▸.

## Supplementary Material

Crystal structure: contains datablock(s) I. DOI: 10.1107/S2056989023007351/mw2198sup1.cif

Structure factors: contains datablock(s) I. DOI: 10.1107/S2056989023007351/mw2198Isup2.hkl

Supporting information file. DOI: 10.1107/S2056989023007351/mw2198Isup3.cml

CCDC reference: 2289922

Additional supporting information:  crystallographic information; 3D view; checkCIF report

## Figures and Tables

**Figure 5:**
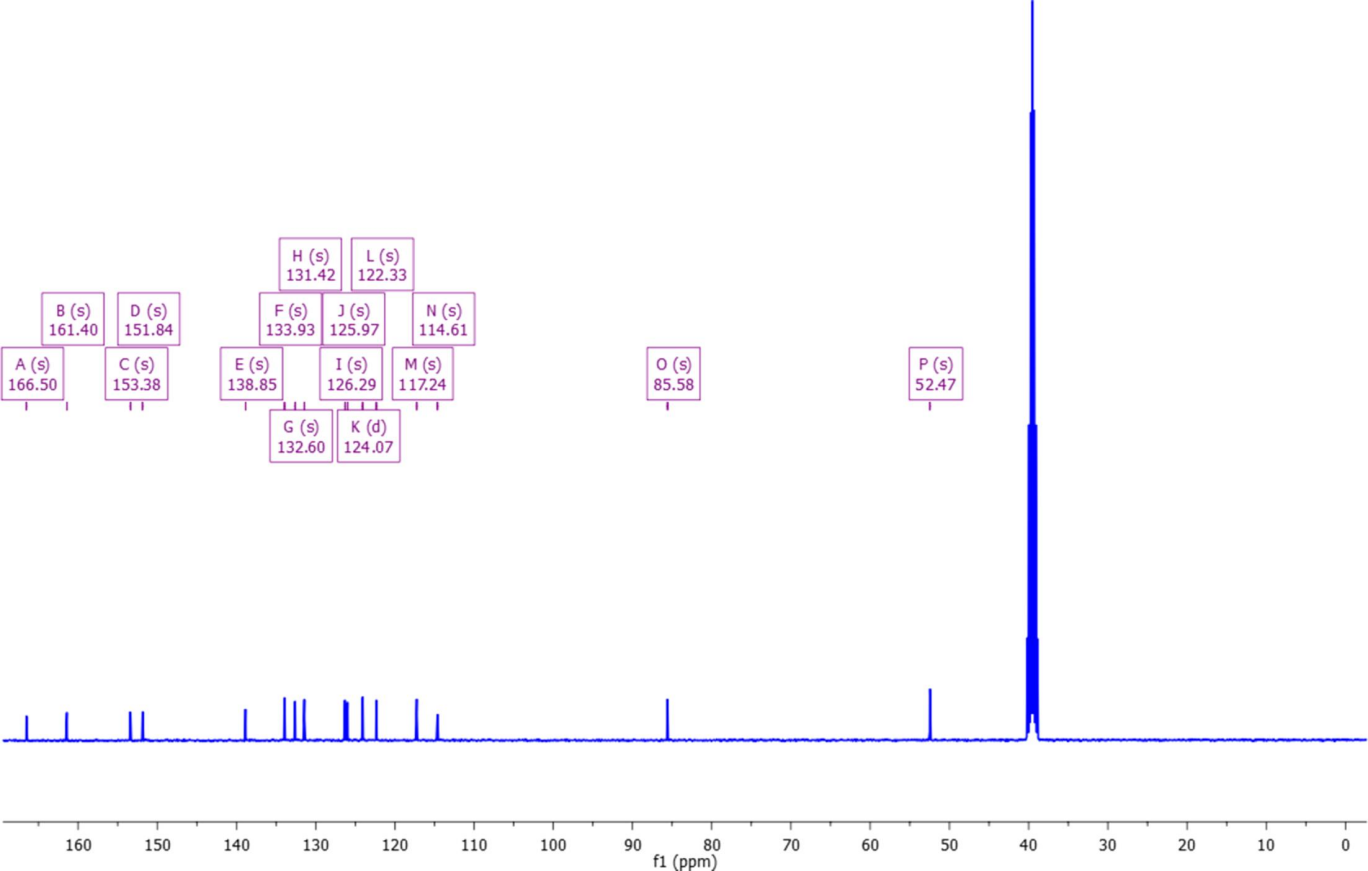
NMR-C: ^13^C NMR (101 MHz, DMSO-*d*_6_) δ 166.50 (*s*) 161.40 (*s*), 153.38 (*s*), 151.84 (*s*), 138.85 (*s*), 133.93 (*s*), 132.60 (*s*), 131.42 (*s*), 126.29 (*s*), 125.97 (*s*), 124.07 (*d*, *J* = 4.0 Hz), 122.33 (*s*), 117.24 (*s*), 114.61 (*s*), 85.58 (*s*), 52.47 (*s*).

**Table 1:** Hydrogen-bond geometry (Å, °)

*D*—H⋯*A*	*D*—H	H⋯*A*	*D*⋯*A*	*D*—H⋯*A*
C1—H1*B*⋯O4^i^	0.96	2.52	3.232 (8)	131
C7—H7⋯O4^ii^	0.93	2.47	3.387 (6)	167
N1—H1⋯O2	0.84 (6)	1.92 (6)	2.631 (6)	141 (5)

Symmetry codes: (i) 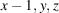 ; (ii) 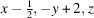 .

**Table 2:** Experimental details

Crystal data	
Chemical formula	C_17_H_13_NO_4_
*M*_r_	295.28
Crystal system, space group	Orthorhombic, *P* *c* *a*2_1_
Temperature (K)	298
*a*, *b*, *c* (Å)	12.7698 (16), 14.9212 (18), 7.1087 (8)
*V* (Å^3^)	1354.5 (3)
*Z*	4
Radiation type	Mo *K*α
μ (mm^−1^)	0.10
Crystal size (mm)	0.39 × 0.08 × 0.06

Data collection	
Diffractometer	Bruker D8 Venture
Absorption correction	Multi-scan (*SADABS*; Krause *et al.*, 2015 ▸)
*T*_min_, *T*_max_	0.666, 0.745
No. of measured, independent and observed [*I* > 2σ(*I*)] reflections	35019, 2328, 2096
*R*_int_	0.102
(sin θ/λ)_max_ (Å^−1^)	0.595

Refinement	
*R*[*F* ^2^ > 2σ(*F* ^2^)], *wR*(*F* ^2^), *S*	0.066, 0.158, 1.08
No. of reflections	2328
No. of parameters	204
No. of restraints	1
H-atom treatment	H atoms treated by a mixture of independent and constrained refinement
Δρ_max_, Δρ_min_ (e Å^−3^)	0.25, −0.37
Absolute structure	Flack *x* determined using 771 quotients [(*I* ^+^) − (*I* ^−^)]/[(*I* ^+^) + (*I* ^−^)] (Parsons *et al.*, 2013 ▸)
Absolute structure parameter	0.2 (7)

Computer programs: *APEX4* (Bruker, 2021 ▸), *SAINT* (Bruker, 2021 ▸), *SHELXT2018* (Sheldrick, 2015*a* ▸) and *SHELXL2018* (Sheldrick, 2015*b* ▸).

## supplementary crystallographic information

### Crystal data

**Table:**

C_17_H_13_NO_4_	*D*_x_ = 1.448 Mg m^−^^3^
*M**_r_* = 295.28	Mo *K*α radiation, λ = 0.71073 Å
Orthorhombic, *P**c**a*2_1_	Cell parameters from 2328 reflections
*a* = 12.7698 (16) Å	θ = 2.1–25.0°
*b* = 14.9212 (18) Å	µ = 0.10 mm^−^^1^
*c* = 7.1087 (8) Å	*T* = 298 K
*V* = 1354.5 (3) Å^3^	Prismatic, yellow
*Z* = 4	0.39 × 0.08 × 0.06 mm
*F*(000) = 616	

### Data collection

**Table:**

Bruker D8 Venture diffractometer	2096 reflections with *I* > 2σ(*I*)
φ and ω scans	*R*_int_ = 0.102
Absorption correction: multi-scan (*SADABS*; Krause *et al.*, 2015)	θ_max_ = 25.0°, θ_min_ = 2.1°
*T*_min_ = 0.666, *T*_max_ = 0.745	*h* = −15→15
35019 measured reflections	*k* = −17→17
2328 independent reflections	*l* = −7→8

### Refinement

**Table:**

Refinement on *F*^2^	Secondary atom site location: difference Fourier map
Least-squares matrix: full	Hydrogen site location: mixed
*R*[*F*^2^ > 2σ(*F*^2^)] = 0.066	H atoms treated by a mixture of independent and constrained refinement
*wR*(*F*^2^) = 0.158	*w* = 1/[σ^2^(*F*_o_^2^) + (0.0648*P*)^2^ + 1.5253*P*] where *P* = (*F*_o_^2^ + 2*F*_c_^2^)/3
*S* = 1.08	(Δ/σ)_max_ < 0.001
2328 reflections	Δρ_max_ = 0.25 e Å^−^^3^
204 parameters	Δρ_min_ = −0.37 e Å^−^^3^
1 restraint	Absolute structure: Flack *x* determined using 771 quotients [(I+)-(I-)]/[(I+)+(I-)] (Parsons *et al.*, 2013)
Primary atom site location: dual	Absolute structure parameter: 0.2 (7)

### Special details

**Table:**

Geometry. All esds (except the esd in the dihedral angle between two l.s. planes) are estimated using the full covariance matrix. The cell esds are taken into account individually in the estimation of esds in distances, angles and torsion angles; correlations between esds in cell parameters are only used when they are defined by crystal symmetry. An approximate (isotropic) treatment of cell esds is used for estimating esds involving l.s. planes.

### Fractional atomic coordinates and isotropic or equivalent isotropic displacement parameters (Å^2^)

**Table:**

	*x*	*y*	*z*	*U*_iso_*/*U*_eq_	
O1	0.4084 (3)	0.8237 (3)	0.3512 (7)	0.0553 (12)	
O2	0.5181 (3)	0.7166 (2)	0.4363 (8)	0.0536 (12)	
O3	1.0000 (3)	0.6344 (3)	0.4928 (7)	0.0527 (12)	
O4	1.0759 (3)	0.7520 (3)	0.3698 (9)	0.0669 (15)	
N1	0.7132 (3)	0.7623 (3)	0.5069 (7)	0.0364 (11)	
H1	0.664 (4)	0.725 (4)	0.502 (8)	0.032 (15)*	
C1	0.3280 (5)	0.7569 (5)	0.3295 (12)	0.067 (2)	
H1A	0.349617	0.713581	0.237447	0.101*	
H1B	0.264282	0.785039	0.288804	0.101*	
H1C	0.316334	0.727407	0.447724	0.101*	
C2	0.5005 (4)	0.7946 (4)	0.4076 (9)	0.0405 (13)	
C3	0.5768 (4)	0.8683 (3)	0.4321 (8)	0.0367 (13)	
C4	0.6811 (4)	0.8507 (3)	0.4842 (8)	0.0326 (11)	
C5	0.7468 (4)	0.9226 (3)	0.5221 (8)	0.0387 (13)	
H5	0.814613	0.912215	0.564520	0.046*	
C6	0.7126 (5)	1.0084 (4)	0.4974 (10)	0.0477 (15)	
H6	0.758040	1.055801	0.520819	0.057*	
C7	0.6125 (5)	1.0258 (3)	0.4388 (11)	0.0539 (17)	
H7	0.590562	1.084558	0.419550	0.065*	
C8	0.5454 (4)	0.9566 (4)	0.4089 (10)	0.0476 (15)	
H8	0.476977	0.968658	0.372170	0.057*	
C9	0.8095 (4)	0.7251 (3)	0.5005 (8)	0.0325 (11)	
C10	0.8978 (4)	0.7656 (3)	0.4380 (9)	0.0385 (13)	
H10	0.894045	0.825120	0.399951	0.046*	
C11	0.9952 (4)	0.7211 (4)	0.4284 (10)	0.0469 (15)	
C12	0.9119 (4)	0.5900 (4)	0.5527 (9)	0.0401 (13)	
C13	0.8153 (4)	0.6322 (3)	0.5592 (8)	0.0342 (12)	
C14	0.7308 (4)	0.5799 (3)	0.6151 (8)	0.0371 (13)	
H14	0.664447	0.605621	0.619064	0.045*	
C15	0.7421 (6)	0.4924 (4)	0.6640 (9)	0.0467 (14)	
H15	0.684223	0.458872	0.700802	0.056*	
C16	0.8405 (5)	0.4536 (4)	0.6587 (10)	0.0549 (17)	
H16	0.848964	0.394016	0.694065	0.066*	
C17	0.9249 (5)	0.5021 (4)	0.6022 (11)	0.058 (2)	
H17	0.990900	0.475834	0.597178	0.069*	

### Atomic displacement parameters (Å^2^)

**Table:**

	*U*^11^	*U*^22^	*U*^33^	*U*^12^	*U*^13^	*U*^23^
O1	0.033 (2)	0.047 (2)	0.086 (3)	0.0037 (19)	−0.006 (2)	0.004 (2)
O2	0.034 (2)	0.034 (2)	0.093 (4)	−0.0002 (17)	0.001 (2)	0.001 (2)
O3	0.034 (2)	0.039 (2)	0.085 (3)	0.0077 (16)	−0.006 (2)	−0.011 (2)
O4	0.031 (2)	0.053 (3)	0.117 (4)	−0.009 (2)	0.014 (2)	−0.020 (3)
N1	0.028 (2)	0.024 (2)	0.057 (3)	0.0006 (18)	0.001 (2)	0.004 (2)
C1	0.034 (3)	0.073 (5)	0.095 (6)	−0.006 (3)	−0.002 (4)	0.000 (4)
C2	0.028 (3)	0.038 (3)	0.055 (4)	0.008 (2)	0.008 (3)	−0.001 (3)
C3	0.032 (3)	0.031 (3)	0.046 (3)	0.006 (2)	0.006 (2)	−0.002 (2)
C4	0.035 (3)	0.032 (2)	0.030 (3)	0.005 (2)	0.003 (2)	0.000 (2)
C5	0.035 (3)	0.034 (3)	0.047 (3)	0.000 (2)	−0.002 (3)	−0.002 (3)
C6	0.054 (4)	0.031 (3)	0.058 (4)	−0.004 (2)	0.004 (3)	−0.004 (3)
C7	0.056 (4)	0.025 (3)	0.081 (5)	0.011 (3)	0.005 (4)	0.001 (3)
C8	0.038 (3)	0.038 (3)	0.066 (4)	0.014 (2)	0.003 (3)	0.004 (3)
C9	0.032 (3)	0.028 (2)	0.037 (3)	0.002 (2)	−0.003 (2)	−0.003 (2)
C10	0.035 (3)	0.029 (3)	0.051 (3)	−0.001 (2)	−0.002 (3)	−0.003 (2)
C11	0.031 (3)	0.042 (3)	0.068 (4)	−0.001 (2)	−0.001 (3)	−0.021 (3)
C12	0.034 (3)	0.034 (3)	0.052 (3)	0.003 (2)	−0.009 (3)	−0.007 (3)
C13	0.035 (3)	0.028 (3)	0.040 (3)	0.005 (2)	−0.006 (2)	−0.005 (2)
C14	0.037 (3)	0.029 (3)	0.045 (3)	0.006 (2)	−0.001 (2)	−0.001 (2)
C15	0.057 (4)	0.035 (3)	0.048 (4)	−0.005 (3)	−0.001 (3)	0.002 (3)
C16	0.069 (4)	0.032 (3)	0.064 (4)	0.006 (3)	−0.018 (4)	0.004 (3)
C17	0.046 (3)	0.041 (4)	0.087 (5)	0.021 (3)	−0.024 (4)	−0.007 (3)

### Geometric parameters (Å, º)

**Table:**

O1—C2	1.316 (7)	C6—H6	0.9300
O1—C1	1.440 (8)	C7—C8	1.358 (8)
O2—C2	1.203 (6)	C7—H7	0.9300
O3—C12	1.373 (7)	C8—H8	0.9300
O3—C11	1.374 (7)	C9—C10	1.353 (7)
O4—C11	1.204 (7)	C9—C13	1.450 (7)
N1—C9	1.351 (6)	C10—C11	1.412 (7)
N1—C4	1.391 (6)	C10—H10	0.9300
N1—H1	0.84 (6)	C12—C17	1.367 (8)
C1—H1A	0.9600	C12—C13	1.385 (7)
C1—H1B	0.9600	C13—C14	1.390 (7)
C1—H1C	0.9600	C14—C15	1.359 (7)
C2—C3	1.479 (7)	C14—H14	0.9300
C3—C8	1.387 (7)	C15—C16	1.385 (10)
C3—C4	1.407 (7)	C15—H15	0.9300
C4—C5	1.388 (7)	C16—C17	1.359 (10)
C5—C6	1.363 (8)	C16—H16	0.9300
C5—H5	0.9300	C17—H17	0.9300
C6—C7	1.370 (9)		
			
C2—O1—C1	116.2 (5)	C7—C8—H8	119.3
C12—O3—C11	121.5 (4)	C3—C8—H8	119.3
C9—N1—C4	130.9 (4)	N1—C9—C10	125.8 (5)
C9—N1—H1	114 (4)	N1—C9—C13	115.5 (4)
C4—N1—H1	114 (4)	C10—C9—C13	118.6 (4)
O1—C1—H1A	109.5	C9—C10—C11	122.7 (5)
O1—C1—H1B	109.5	C9—C10—H10	118.7
H1A—C1—H1B	109.5	C11—C10—H10	118.7
O1—C1—H1C	109.5	O4—C11—O3	116.0 (5)
H1A—C1—H1C	109.5	O4—C11—C10	126.3 (6)
H1B—C1—H1C	109.5	O3—C11—C10	117.7 (5)
O2—C2—O1	122.5 (5)	C17—C12—O3	116.3 (5)
O2—C2—C3	125.2 (5)	C17—C12—C13	122.4 (6)
O1—C2—C3	112.3 (4)	O3—C12—C13	121.3 (5)
C8—C3—C4	118.8 (5)	C12—C13—C14	116.5 (5)
C8—C3—C2	120.1 (5)	C12—C13—C9	118.1 (5)
C4—C3—C2	121.1 (4)	C14—C13—C9	125.4 (5)
C5—C4—N1	122.2 (5)	C15—C14—C13	121.9 (6)
C5—C4—C3	118.7 (5)	C15—C14—H14	119.0
N1—C4—C3	119.0 (5)	C13—C14—H14	119.0
C6—C5—C4	120.4 (5)	C14—C15—C16	119.4 (6)
C6—C5—H5	119.8	C14—C15—H15	120.3
C4—C5—H5	119.8	C16—C15—H15	120.3
C5—C6—C7	121.1 (5)	C17—C16—C15	120.3 (6)
C5—C6—H6	119.5	C17—C16—H16	119.8
C7—C6—H6	119.5	C15—C16—H16	119.8
C8—C7—C6	119.4 (5)	C16—C17—C12	119.4 (6)
C8—C7—H7	120.3	C16—C17—H17	120.3
C6—C7—H7	120.3	C12—C17—H17	120.3
C7—C8—C3	121.5 (5)		
			
C1—O1—C2—O2	1.4 (10)	C13—C9—C10—C11	−0.2 (9)
C1—O1—C2—C3	−178.0 (6)	C12—O3—C11—O4	−176.1 (6)
O2—C2—C3—C8	−174.9 (7)	C12—O3—C11—C10	4.4 (9)
O1—C2—C3—C8	4.5 (8)	C9—C10—C11—O4	177.8 (7)
O2—C2—C3—C4	3.2 (9)	C9—C10—C11—O3	−2.7 (9)
O1—C2—C3—C4	−177.3 (5)	C11—O3—C12—C17	175.9 (6)
C9—N1—C4—C5	−26.1 (9)	C11—O3—C12—C13	−3.2 (8)
C9—N1—C4—C3	157.9 (6)	C17—C12—C13—C14	−1.4 (9)
C8—C3—C4—C5	4.0 (8)	O3—C12—C13—C14	177.6 (6)
C2—C3—C4—C5	−174.2 (6)	C17—C12—C13—C9	−178.9 (6)
C8—C3—C4—N1	−179.7 (6)	O3—C12—C13—C9	0.1 (8)
C2—C3—C4—N1	2.1 (8)	N1—C9—C13—C12	179.0 (5)
N1—C4—C5—C6	179.8 (6)	C10—C9—C13—C12	1.6 (8)
C3—C4—C5—C6	−4.2 (9)	N1—C9—C13—C14	1.7 (8)
C4—C5—C6—C7	1.4 (10)	C10—C9—C13—C14	−175.7 (5)
C5—C6—C7—C8	1.6 (11)	C12—C13—C14—C15	1.1 (8)
C6—C7—C8—C3	−1.6 (11)	C9—C13—C14—C15	178.4 (6)
C4—C3—C8—C7	−1.2 (10)	C13—C14—C15—C16	0.1 (9)
C2—C3—C8—C7	177.0 (7)	C14—C15—C16—C17	−1.1 (10)
C4—N1—C9—C10	−11.9 (10)	C15—C16—C17—C12	0.8 (10)
C4—N1—C9—C13	171.0 (6)	O3—C12—C17—C16	−178.6 (6)
N1—C9—C10—C11	−177.3 (6)	C13—C12—C17—C16	0.5 (10)

### Hydrogen-bond geometry (Å, º)

**Table:**

*D*—H···*A*	*D*—H	H···*A*	*D*···*A*	*D*—H···*A*
C1—H1*B*···O4^i^	0.96	2.52	3.232 (8)	131
C7—H7···O4^ii^	0.93	2.47	3.387 (6)	167
N1—H1···O2	0.84 (6)	1.92 (6)	2.631 (6)	141 (5)

Symmetry codes: (i) *x*−1, *y*, *z*; (ii) *x*−1/2, −*y*+2, *z*.
